# Supplementary material for: Weizmannia coagulans BC2000 Plus Ellagic Acid Inhibits High-Fat-Induced Insulin Resistance by Remodeling the Gut Microbiota and Activating the Hepatic Autophagy Pathway in Mice
Source: Nutrients. 2022 Oct 9;14(19):4206. doi: 10.3390/nu14194206 (PMC9572659; doi:10.3390/nu14194206)
Supplement: Supplementary file 1 [file nutrients-14-04206-s001.zip › nutrients-1930135-supplementary.pdf]

Supplementary Information

1. Supplementary Methods

1.1 Evaluation of the Probiotic Properties of *Weizmannia. Coagulans*

1.1.1 Sample information

The strains used in this experiment are *Weizmannia coagulans* BC2000 (No. # 1), a Chinese brand strain (No. # 2), a Chinese brand strain (No. # 3), a Chinese brand strain (No. # 4), a Taiwanese brand strain (No. # 5) and a brand of strain from a country except China (No. # 6). All 6 samples were *Weizmannia coagulans.*

1.1.2 *Weizmannia coagulans* heat resistance test

Six samples of *Weizmannia coagulans* were configured into bacterial suspensions using saline at approximately $1.0*10^{10}$ CFU/mL. The suspensions were first placed in a water bath at 80°C for 10 min as a control. The control suspensions were then placed in a water bath at 80°C and 90°C for 30 min and counted.

1.1.3 *Weizmannia coagulans* acid resistance test

Six samples of *Weizmannia coagulans* were configured into bacterial suspensions using saline at approximately $1.0*10^{10}$ CFU/mL. The suspensions were first kept in a water bath at 80°C for 10 min as a control. Dipotassium hydrogen phosphate and citric acid were added to give a final concentration of 1.76 g/L and 1.27 g/L respectively, and then the suspension was adjusted to pH 2.0 with 1 mol/L hydrochloric acid and left at 37°C for 2 h before counting.

1.1.4 *Weizmannia coagulans* bile salt resistance test

Six samples of *Weizmannia coagulans* were configured into bacterial suspensions using saline at approximately $1.0*10^{10}$ CFU/mL. The suspensions were first kept in a water bath at 80°C for 10 min as a control. The control suspensions were then spiked with 0.03% and 0.3% sodium bile acids, respectively, and left at 37°C for 24 h before counting.

2. Supplementary Figure

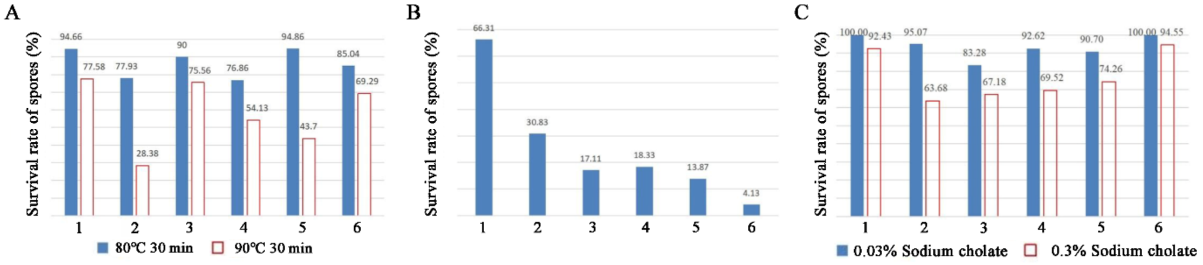

Figure S1. Probiotic properties of *Weizmannia coagulans*. (A) Heat resistance; (B) Acid resistance; (C) Bile salt resistance. No. # 1 means *Weizmannia coagulans* BC2000; No. # 2 means a Chinese brand of strain; No. # 3 means a Chinese brand of strain; No. # 4 means a Chinese brand of strain; No. # 5 means a Taiwanese brand strain and No. # 6 means a brand of strain from a country except for China. All 6 samples were *Weizmannia coagulans*.
